# Intraspecific sequence variation and differential expression in starch synthase genes of *Arabidopsis thaliana*

**DOI:** 10.1186/1756-0500-6-84

**Published:** 2013-03-06

**Authors:** Sandra Schwarte, Henrike Brust, Martin Steup, Ralph Tiedemann

**Affiliations:** 1 Evolutionary Biology, Institute of Biochemistry and Biology, University of Potsdam, Karl-Liebknecht-Strasse 24-25, Building 26, Potsdam, 14476, Germany; 2 Plant Physiology, Institute of Biochemistry and Biology, University of Potsdam, Karl-Liebknecht-Strasse 24-25, Building 20, Potsdam, 14476, Germany

**Keywords:** *Arabidopsis thaliana*, Starch synthases, Genetic variation, Transcript level

## Abstract

**Background:**

Natural accessions of *Arabidopsis thaliana* are a well-known system to measure levels of intraspecific genetic variation. Leaf starch content correlates negatively with biomass. Starch is synthesized by the coordinated action of many (iso)enzymes. Quantitatively dominant is the repetitive transfer of glucosyl residues to the non-reducing ends of α-glucans as mediated by starch synthases. In the genome of *A*. *thaliana*, there are five classes of starch synthases, designated as soluble starch synthases (SSI, SSII, SSIII, and SSIV) and granule-bound synthase (GBSS). Each class is represented by a single gene. The five genes are homologous in functional domains due to their common origin, but have evolved individual features as well. Here, we analyze the extent of genetic variation in these fundamental protein classes as well as possible functional implications on transcript and protein levels.

**Findings:**

Intraspecific sequence variation of the five starch synthases was determined by sequencing the entire loci including promoter regions from 30 worldwide distributed accessions of *A*. *thaliana*. In all genes, a considerable number of nucleotide polymorphisms was observed, both in non-coding and coding regions, and several amino acid substitutions were identified in functional domains. Furthermore, promoters possess numerous polymorphisms in potentially regulatory *cis*-acting regions. By realtime experiments performed with selected accessions, we demonstrate that DNA sequence divergence correlates with significant differences in transcript levels.

**Conclusions:**

Except for *AtSSII*, all starch synthase classes clustered into two or three groups of haplotypes, respectively. Significant difference in transcript levels among haplotype clusters in *AtSSIV* provides evidence for *cis*-regulation. By contrast, no such correlation was found for *AtSSI*, *AtSSII*, *AtSSIII*, and *AtGBSS*, suggesting *trans*-regulation. The expression data presented here point to a regulation by common *trans*-regulatory transcription factors which ensures a coordinated action of the products of these four genes during starch granule biosynthesis. The apparent *cis*-regulation of *AtSSIV* might be related to its role in the initiation of *de novo* biosynthesis of granules.

## Background

*Arabidopsis thaliana* accessions are naturally occurring and essentially homozygous inbred lines that are frequently used to investigate genetic and/or metabolic variations [1-4] and to identify genes relevant for intraspecific adaptation phenomena in plants [5-7]. Due to intraspecific genetic variation, many *A*. *thaliana* accessions differ in growth and development even when grown alongside under the same conditions [8,9]. Since their divergence from *A*. *lyrata* 5-10 million years ago, *A*. *thaliana* accessions possess a long evolutionary history of intraspecific diversification [10,11]. Genetic variation leads to nucleotide polymorphisms in both coding and noncoding gene regions. Nonsynonymous substitutions locally alter the amino acid sequence of either the transit peptide or the mature protein at the level of translation and, thereby, potentially may affect protein-related functions. Synonymous substitutions do not alter the amino acid sequence but may affect level and/or stability of the transcripts as well as the rate of translation. Thereby, they might indirectly alter the level of a given protein. Likewise, variation in noncoding regions is not translated into amino acid polymorphisms, but can exert diverse effects, such as alternative splicing, introduction of premature stop codons of transcription or translation, altered transcripts stability and/or rate of gene expression [12]. Thereby, it indirectly may also affect level and/or amino acid sequence of a given protein.

To a large extent, regulation of gene expression is based on the action of regulatory elements that are located in positions designated as *cis* (i.e. close to the target gene) or *trans* (i.e. distant from the gene) [13,14]. In summary, genetic diversity may affect phenotypic traits by acting on different levels ranging from gene expression to transcript level and altered features and/or functions of the protein.

In many *A*. *thaliana* accessions studied so far, vegetative biomass is negatively correlated with leaf starch content [15]. Therefore, intraspecific genetic variation appears to massively affect the central carbon metabolism and growth of the entire plant. In *A*. *thaliana*, as in many other plant species, transitory starch is a major product of photosynthesis which is deposited in the stromal space of the mesophyll chloroplasts as water-insoluble particles (i.e. granules). They possess a strictly defined and evolutionary conserved (inter)molecular order [16,17] and consist of two types of polyglucans, amylopectin and amylose. Amylopectin is a large, highly branched polyglucan representing the main constituent of the granule. By contrast, amylose is a polydispers and essentially unbranched biopolymer that, in most cases, is a minor starch compound and contributes little to the (inter)molecular organization of the entire starch particle [17].

Starch biochemistry is based on the coordinated and evolutionary conserved action of 30 to 40 (iso)enzymes and is more complex than the classical glycogen metabolism [18]. Massive starch biosynthesis proceeds by a repetitive glucosyl transfer from an appropriate donor (such as ADPglucose) to non-reducing ends of oligo- or polyglucans [19]. ADPglucose-dependent chain elongation is mediated by at least five classes of starch synthases (ADP-Glc: α-1,4 glucan α-4-glucosyl transferase; EC 2.4.1.21). In *A*. *thaliana*, each class is represented by only a single gene. Based on sequence similarity, kinetic properties, and the occurrence of consensus motifs, they are all related to the glycogen synthases from both prokaryotes and eukaryotes and are members of the glucosyl transferase family 5 (GT5).

The five starch synthase classes comprise four soluble synthases (SSI to SSIV) and one granule-bound starch synthase (GBSS). The five classes are conserved in green algae and higher plant species (Table 1). Soluble starch synthases (SS) occur in the stromal space of plastids, but a proportion is often found tightly associated with native starch. By contrast, granule-bound starch synthase (GBSS) is essentially entirely integrated into the starch granules [20]. Most SS classes catalyze distinct steps within the amylopectin biosynthesis [17,21]. *A*. *thaliana* mutants in which a single SS class is not functional show specific starch-related phenotypes (such as alterations in the number and/or the size of starch granules or the chain length pattern within the amylopectin molecules). Because of the distinct phenotype of these mutants, SS classes are unlikely to possess fully redundant functions *in vivo*, but exhibit class-specific features (subfunctionalization) [11,17,21].

**Table 1:** Number of genes encoding each starch synthase class in different plant species

	**SSI**	**SSII**	**SSIII**	**SSIV**	**GBSS**	**references**
***Chlamydomonas reinhardtii***	2	1	2	1	2	[22]
***Volvox carteri***	1	2	1	2	1	[22]
***Ostreococcus tauri***	1	1	3	-	1	[22]
***Ostreococcus lucimarius***	1	1	3	1	1	[22]
***Arabidopsis thaliana***	1	1	1	1	1	[22]
***Solanum tuberosum***	1	1	1	-	1	[18,23]
***Zea mays***	1	2	1	1	1	[18]
***Oryza sativa***	1	3	2	2	2	[18,23,24]

Unlike in *A*. *thaliana*, in some lower and higher plants starch synthase classes are represented by more than a single gene. Complexity of the starch synthase classes tends to increase if cells or tissues are capable of metabolizing various starch pools (such as transitory and reserve starches) that are spatially and/or temporarily separated (Table 1).

The five SS classes (and the glycogen synthases as well) share a core region of approximately 60 kDa that is indispensable for catalytic activity and consists of two conserved domains often designated as GT5 (glycosyl transferase family 5) and GT1 (glycosyl transferase family 1). Both domains are separated by a short and more variable linker region. The GT5 domain is typical for glucosyl transferases following a retaining mechanism [25]. GT1 is consistently located close to the C-terminus of the starch/glycogen synthases and is frequently found in glycosyl transferases mediating an inverting mode of glycosyl transfer [26]. In all starch synthases, both the GT5 and GT1 domain are involved in binding of the glucosyl donor, ADPglucose, and together they form the catalytically active region of starch synthases.

**Figure 1:**
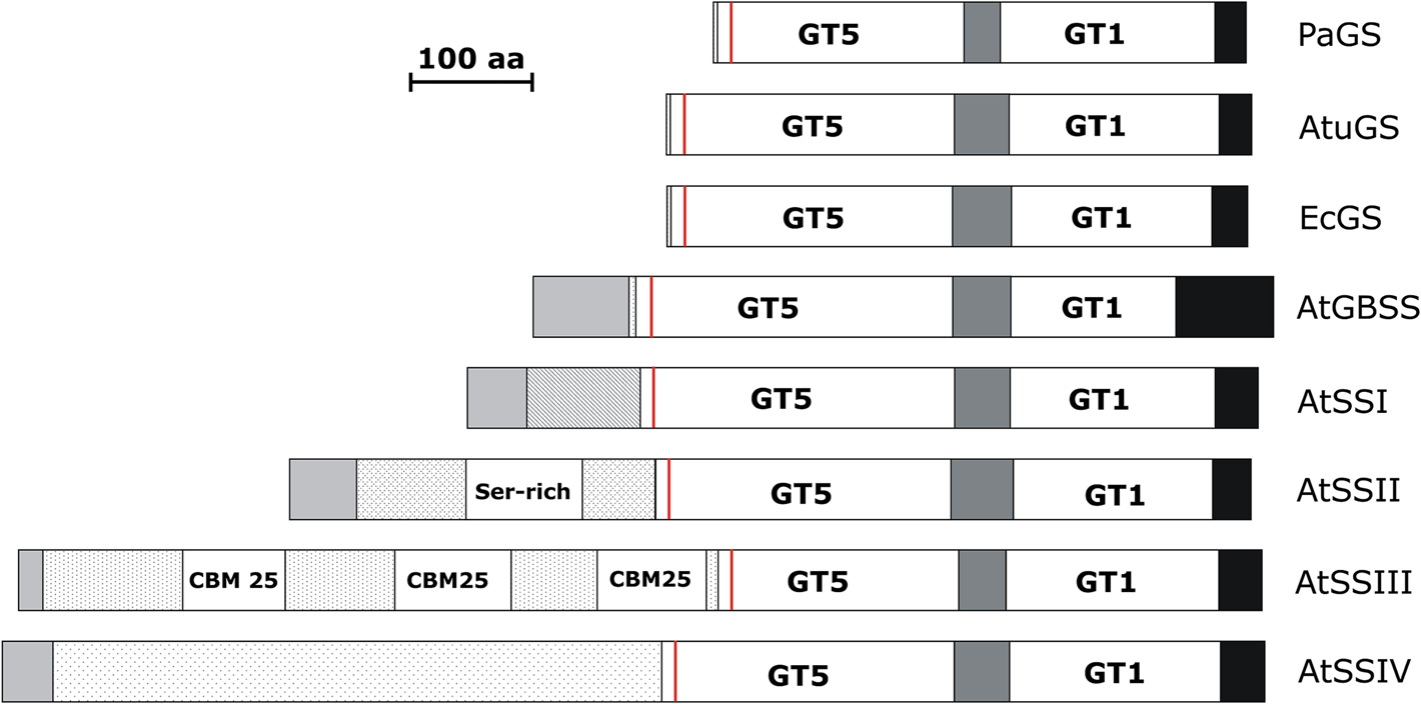
**Domain structure of the five starch synthase proteins from *A. thaliana*.** The starch synthases from *A. thaliana* are the granula-bound starch synthase (AtGBSS) and four soluble synthases (AtSSI to AtSSIV). For comparison, three prokaryotic glycogen synthases [from *Pyrococcus abyssi* (PaGS), *Agrobacterium tumefaciens* (AtuGS) and *Escherichia coli* (EcGS)] were included. The N-terminus of the starch/glycogen synthases is at the left, the C-terminus (black) is at the right. The conserved domains of the glycosyl transferase family 5 (GT5) and the glycosyl transferase family 1 (GT1) are given in white. The linker region between GT5 and GT1 (dark gray) and C-terminal extensions (black) are indicated. The red line at the N-terminal region of GT5 marks the position of the highly conserved motif KXGGL. The N-terminal extension of AtSSII contains a serine-rich (Ser-rich) region and three conserved carbohydrate binding modules of family 25 (CBM 25). For all starch synthases, the N-terminal transit peptides are given in grey.

A scheme of the domain structure of the five *A*. *thaliana* starch synthases and of three prokaryotic glycogen synthases is presented in Figure 1. AtGBSS largely consists of the core region containing the two domains GT5 and GT1 and possesses only a short additional sequence at the C-terminus. The size of AtGBSS is similar to that of the prokaryotic glycogen synthases. All soluble starch synthases (AtSSI to AtSSIV) carry a N-terminal extension that might modulate catalytic activity. The length of the extension strongly varies both among SS classes of a given plant species and between species. A unique feature of the N-terminal sequence of AtSSII is a serine-rich region of uncertain function. AtSSIII and AtSSIV possess large N-terminal extensions that are similar in size, but diverse in amino acid sequence. The N-terminal sequence of AtSSIII contains three repeats of a distinct carbohydrate binding module (CBM25) [27,28] that are not present in AtSSIV (Figure 1).

In previous studies, *AtSSI* has been localized in one of the few genome regions that possess a high intraspecific variability [29]. Here, we used 30 *A*. *thaliana* accessions and sequenced genes encoding the five starch synthases including their promoter regions. Accessions were selected on the basis of both largely different climates at their original habitats and wide geographic distribution of the natural populations. The objective was to estimate the gene-specific level of variation as well as possible implications for gene expression and protein structure. Specifically, we were interested in: (i) whether genes of the five starch synthase classes exhibit a similar degree of both synonymous and nonsynonymous variation, (ii) whether specific gene trees of the five starch synthase classes show the same pattern of haplotype clustering across accessions, (iii) whether selection can be inferred to act on genes and/or single polymorphic sites, (iv) whether polymorphisms across accessions and starch synthase classes have functional implications, and (v) whether there is a relationship between genomic variation and transcript levels in starch synthases.

## Findings

### Nucleotide polymorphisms in genes of the five starch synthase classes

For 30 *A*. *thaliana* accessions, genes encoding four soluble starch synthases (*AtSSI*: *At5g24300*; *AtSSII*: *At3g01180*; *AtSSIII*: *At1g11720*, and *AtSSIV*: *At4g18240*) and the granule bound starch synthase (*AtGBSS*: *At1g32900*) were sequenced and analyzed regarding intra- and interspecific genetic variation (as compared to *A*. *lyrata*). In all starch synthase genes, coding regions have higher GC contents (40–45%; Table 2) than noncoding regions (30–35%), a general feature of eukaryotic genes [30]. The relative frequency of nonsynonymous substitutions ranged from 0.46% (*AtSSI*) to 1.10% (*AtSSIII*).

**Table 2:** **Sequence comparison of starch synthases from 30 accessions of *A. thaliana***

	**Domain**	**Sites**	***S***	**η**	**Nonsyn**	**Indels**	***h***	***Hd***	**π**	**GC content**
***AtSSI***	gene	3946	126	128	9	26	21	0.966	0.0112	0.396
	exons	1959	30	30	9	-	8	0.584	0.0047	0.467
	introns	1987	96	98	-	26	21	0.966	0.0180	0.321
	promoter	1422	68	71	-	24	22	0.972	0.0151	0.309
	cTP	147	2	2	0	-	2	0.405	0.0055	0.515
	GT5	780	8	8	4	-	4	0.499	0.0024	0.470
	GT1	510	13	13	2	-	3	0.421	0.0097	0.474
***AtSSII***	gene	3226	31	31	13	3	12	0.841	0.0014	0.423
	exons	2379	19	19	13	-	10	0.782	0.0012	0.451
	introns	847	12	12	-	3	7	0.611	0.0019	0.341
	promoter	855	26	26	-	13	14	0.913	0.0063	0.304
	cTP	165	4	4	3	-	3	0.131	0.0016	0.491
	GT5	732	8	8	4	-	8	0.749	0.0025	0.444
	GT1	495	0	0	0	-	1	0.000	0.0000	0.467
***AtSSIII***	gene	4358	105	106	34	11	17	0.929	0.0056	0.403
	exons	3099	63	64	34	1	14	0.899	0.0047	0.422
	introns	1259	42	42	-	10	10	0.811	0.0078	0.357
	promoter	937	8	8	-	4	7	0.676	0.0016	0.356
	cTP	60	0	0	0	-	1	0.000	0.0000	0.517
	GT5	597	15	15	6	-	6	0.680	0.0057	0.406
	GT1	528	11	11	2	-	3	0.297	0.0047	0.444
***AtSSIV***	gene	4874	72	73	17	15	23	0.977	0.0028	0.379
	exons	3123	31	31	17	-	15	0.839	0.0018	0.413
	introns	1751	41	42	-	15	18	0.952	0.0047	0.316
	promoter	547	30	30	-	8	12	0.834	0.0125	0.366
	cTP	126	2	2	2	-	3	0.393	0.0033	0.443
	GT5	726	3	3	2	-	4	0.251	0.0004	0.429
	GT1	525	2	2	1	-	3	0.246	0.0005	0.411
***AtGBSS***	gene	2989	53	53	12	10	17	0.945	0.0045	0.403
	exons	1833	28	28	12	-	14	0.857	0.0042	0.452
	introns	1156	25	25	-	10	16	0.903	0.0050	0.319
	promoter	906	37	38	-	11	15	0.894	0.0078	0.356
	cTP	237	15	15	9	-	5	0.499	0.0223	0.406
	GT5	786	7	7	3	-	8	0.630	0.0017	0.442
	GT1	411	4	4	0	-	5	0.575	0.0019	0.486

Promoter = either the complete intergenic region or about 1 kb upstream the coding region; cTP = chloroplast transit peptide; GT5 domain = starch synthase catalytic domain; GT1 domain = glucosyl transferase group 1; *S* = polymorphic sites; η = total number of mutations; nonsyn *=* nonsynonymous sites; indels = number of insertions/deletions; *h =* number of haplotypes (=alleles); *Hd =* haplotype diversity; π = nucleotide diversity.

**Figure 2:**
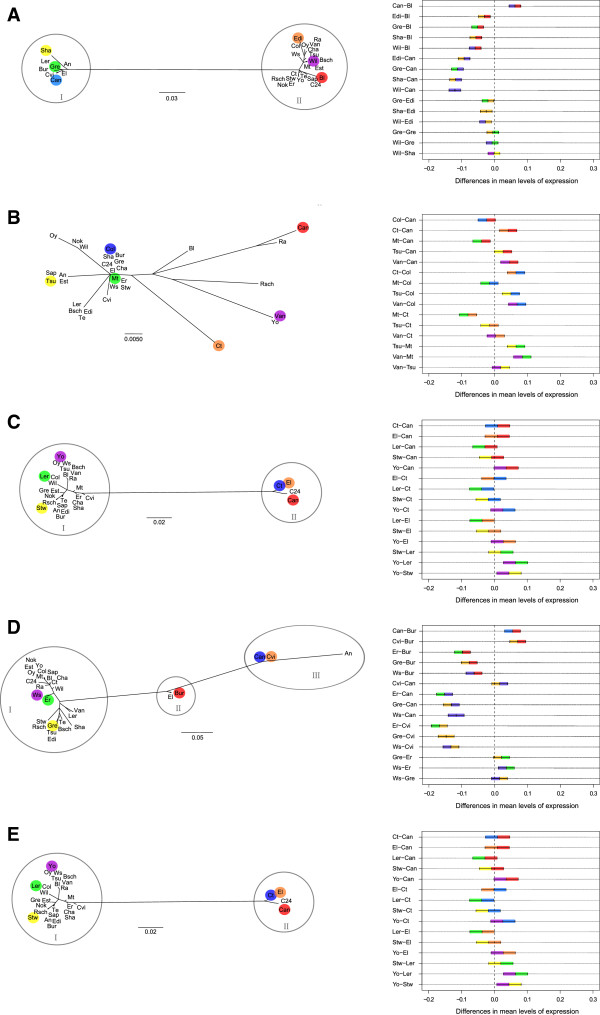
**Clustering of *A. thaliana *accessions and their corresponding transcript levels. A**: *AtSSI*; **B**: *AtSSII*; **C**: *AtSSIII*; **D**: *AtSSIV*; **E**: *AtGBSS*. Left: Unrooted maximum likelihood tree among accessions based on promoter and gene sequences. The clusters for each gene, except *AtSSII*, are highlighted with circles. Right: TukeyHSD test for verification of significant differences in transcript levels among accessions. Six accessions representing different clusters of respective starch synthase were used for Realtime PCR analysis and are marked with the same color in the left and right panel. Pairwise comparisons of transcript levels with confidence levels of 95% that are different from zero indicate significant differences in transcript levels among accessions.

Among the accessions studied, *AtSSI* possesses the highest overall nucleotide diversity. Substitutions are unevenly distributed along the gene, as most of them occur between position 2,300 and 3,700 (Additional file 1: Figure S1A). The majority of substitutions are found in a distinct subset of accessions (An, Bur, Can, Cvi, El, Gre, Ler, and Sha; Additional file 1: Figure S2A) which form a separate cluster in the *AtSSI* gene tree (Figure 2A). *AtSSII* shows a lower degree of nucleotide diversity (Table 2) with most substitutions being located between position 1,100 and 2,200 (Additional file 1: Figure S1B). Unlike *AtSSI*, there was no division into haplogroups (Figure 2B, Additional file 1: Figure S2B), as substitutions occur randomly across accessions. *AtSSIII* possesses the highest number of polymorphisms (Table 2), most of which are observed between position 1,000 and 4,300 (Additional file 1: Figure S1C). They exist in the same subset of accessions (C24, Can, Ct, and El; Additional file 1: Figure S2C) which form cluster II in the gene tree (Figure 2C). However, the clustering of *AtSSIII* deviates from that of *AtSSI* (Figure 2A) with regard to the assignment of accessions. Furthermore, we observed a 21 bp indel (= 7 amino acids) in the coding region (exon 1) of C24, Can, Ct, and El that is also present in the closest relative *A*. *lyrata*. *AtSSIV* exhibits an intermediate degree of nucleotide diversity (Table 2, Additional file 1: Figure S1D). Accessions can be assigned to cluster I or III by means of their substitution pattern within the gene (Figure 2D). However, when sequences of the promoter region and the gene are combined, an additional intermediate haplotype arises (cluster II; Figure 2D). In *AtGBSS* highly diverse regions are found both between position 1 to 300, encoding the transit peptide, and in the sequence in close N-terminal vicinity of domain GT5 (Additional file 1: Figure S1E). Many of these substitutions are found in the two haplogroups as inferred by phylogenetic analysis (cluster I and II; Figure 2E; Additional file 1: Figure S2E).

The interspecific diversity (as determined by comparing the analyzed *A*. *thaliana* accessions and *A*. *lyrata*) vastly exceeds the intraspecific diversity. The two *Arabidopsis* species are clearly separated evolutionary lineages, although accessions represent individual haplotypes that are more alike the published *A*. *lyrata* sequence than the *A*. *thaliana* reference Columbia-0 (Additional file 1: Figure S2).

### Amino acid substitutions

We inferred numerous amino acid substitutions in starch synthase genes (Tables 2 and 3; Additional file 1: Figure S2). Some of these substitutions are located in functionally essential regions, i.e., transit peptides and catalytical core regions (Table 2, Additional file 1: Figure S2). In order to classify nonsynonymous substitutions with respect to protein functions, we considered the affected amino acids and checked for conservation of this position among plant species.

**Table 3:** **Nonsynonymous substitutions in starch synthases of *A. thaliana *accessions**

	**Nonsynonymous substitutions**
***AtSSI***	S57F, Q89ED, A191T, K309N, **E326D**, **P327S**, S506N, T584A
***AtSSII***	S29F, H34P, P37A, I138M, D197Y, V198E, E290V, **S329A**, M369T, **F374Y**, S392R, R765T, T769S
***AtSSIII***	N68D, D92N, M197I, T279I, **R321T**, T326A, N352D, **G363R**, F392V, L393M, G398S, Q408R, L410I, N421D, R425K, D430E, **R431K**, M438T, **E451K**, L484F, G502V, T522A, E525GV, I571F, V616I, I623L, **V671I**, F697Y, Q722H, A773P, H779Y, **I844V**, **D875E**
***AtSSIV***	I18F, P34H, I67F, L84P, I144V, A146T, I150V, K156Q, I180V, N228S, G310S, I377L, L499I, I516T, E604D, Q767H, **H857L**
***AtGBSS***	N9H, H20Q, V28L, A29S, G35A, N51K, S66L, R68G, V72G, V140I, M256I, **F291L**

Protein sequences were compared to those from *Oryza sativa*, *Zea mays*, *Solanum tuberosum*, *Populus trichocarpa*, *Hordeum vulgare*, *Phaseolus vulgaris*, *Triticum aestivum* and *Physcomitrella patens* (sequences available in Genbank). Substitutions among *A. thaliana* accessions which affect positions that are usually highly conserved among plant species are printed bold.

#### Transit peptide

Starch synthases are posttranslationally imported into the plastid and possess an N-terminal transit peptide whose size considerably varies between the five classes, *AtSSI*: 49 amino acids (aa), *AtSSII*: 55 aa, *AtSSIII*: 20 aa, *AtSSIV*: 42 aa, *AtGBSS*: 79 aa [31]. Among the analyzed accessions, neither *AtSSI* nor *AtSSIII* have any alterations in the amino acid sequence of the transit peptide, but the three other classes possess several nonsynonymous substitutions (ns): 3 ns in *AtSSI*, 2 ns in *AtSSIV*, and 9 ns in *AtGBSS*.

#### Catalytic core region

In all five starch synthases, nonsynonymous substitutions occur in the GT5 domain (Tables 2 and 3). They are less frequent in *AtSS1* as only two accessions are affected (A191T: Ws; K309N, E326D, P327S: Bl; Table 3). K309N is situated in the α_3_β_8_-loop of the secondary protein structure [32]. In SSI from different plant species, this loop has been reported to contain either arginine or lysine residues. In accession Bl, E326D at position 326 was found. As revealed by an interspecific comparison, at this position glutamic acid is highly conserved among higher plants SSI. However, glutamic acid and aspartic acid have similar biophysical properties [33]. Another nonsynonymous substitution in this accession, P327S, is putatively affecting the secondary structure of AtSSI [33]. As revealed by interspecies sequence comparison, this position is highly conserved for proline [33]. In *AtSSII*, several accessions possess nonsynonymous substitutions in the GT5 domain, all of which are shared among several accessions analyzed (S329A: Bl, Can, Cvi, Ra, Rsch, Van, Yo; M369T: Rsch, Van, Yo; F374Y: Can, Ra, Van, Yo; S392R: Bsch, Edi, Ler, Ra, Te; Table 3). Sequence comparisons between different higher plant species have shown that in SSII alanine is common at position S329A but serine is unusual. M369T is situated in the β_5_-sheet (EVMYFHA) [32] and this position is not conserved in SSII isoforms among higher plants. In F374Y tyrosine is common in SSII isoforms, while phenylalanine is rarely found. S392R is an alteration frequently observed among plant species. However, analyses of the starch synthase IIa in maize revealed that specific arginine residues are important for both protein stability and the interaction with the glucosyl acceptor [23]. The GT5 domain of *AtSSIII* exhibits several polymorphisms (Table 3) that all co-occur in the accessions C24, Can, Ct, and El (V616I, I623L, V671I, Q722H). A further substitution (F697Y) is restricted to Yo. Typically, dicotyls (*Solanum tuberosum*, and *Solanum lycopersicum*) exhibit a phenylalanine residue at this position, while monocotyls (*Oryza sativa*, and *Zea mays*) have serine. Tyrosine, as in the accession Yo, is a rare residue at this position. Busi et al. [34] described by comparative analysis between glycogen synthases of *Agrobacterium tumefaciens* and *ATSSIII* specific residues that are involved in the binding of ADP-glucose and glycogen/starch-derived α-glucan chains. All residues are conserved in the analyzed accessions. In the GT5 domain of *AtSSIV*, two substitutions exist that are observed in only a single accession (E604D: Bur, Q767H: Gre; Table 3). Both sites are known to be variable among plant species. In *AtGBSS*, the GT5 domain exhibits three polymorphisms (Table 3). V140I (in Tsu) is located in an otherwise conserved region.

In the GT1 domain of *AtSSI*, *AtSSIII*, and *AtSSIV*, amino acid sequence variation occurs less frequently. In *AtSSI*, we identified only two nonsynonymous substitutions (S506N: Sha; T584A: An, Bur, Can, Cvi, El, Gre, Ler, Sha). Both positions are variable among higher plant species (Table 3). The GT1 domain from *AtSSIII* exhibits two nonsynonymous substitutions (I844V: Rsch, D875E: Can, Ct, El, C24). As revealed by interspecific comparison, both positions are highly conserved for isoleucine and for aspartic acid, respectively. The GT1 domain from *AtSSIV* is affected by a single nonsynonymous substitution H857L. This exchange is restricted to accession Tsu and is unusual among plants. *AtSSII* and *AtGBSS* possess no polymorphisms in the GT1 domain.

In summary, GT5 is more variable than GT1 regarding the number of both nonsynonymous substitutions and accessions affected. Most amino acid substitutions exist at sites known to be variable but we also identified several unusual substitutions at otherwise highly conserved sites.

### Signs of selection

All tests of gene-wise selection for *AtSSI*, *AtSSIII*, *AtSSIV*, and *AtGBSS* revealed a statistically significant pattern for purifying selection acting on each starch synthase (Table 4). Purifying selection for *AtSSII* was not significantly supported and, therefore, the null hypothesis of neutral evolution cannot be statistically rejected. However, the statistical power of this particular test is limited because of the very low diversity in the coding region of *AtSSII* (π = 0.12%; Table 2). We also calculated Tajima’s *D*, a commonly used selection test often applied in *A*. *thaliana*, but obtained no significant support for selection. Furthermore, we searched for positively selected sites (PSS) using PAML but no nonsynonymous substition was inferred to be under positive selection.

**Table 4:** **Selection tests of starch synthases in *A. thaliana***

	**Positive selection**		**Purifying selection**		**Tajima’s *D***	
	***Z *statistic**	**p value**	***Z *statistic**	**p value**	***D *value**	**p value**
***AtSSI***	−4.150	1.000	4.244	0.000	0.757	p > 0.10
***AtSSII***	−0.706	1.000	0.721	0.236	−1.386	p > 0.10
***AtSSIII***	−2.870	1.000	2.811	0.003	−0.357	p > 0.10
***AtSSIV***	−1.692	1.000	1.758	0.041	−0.992	p > 0.10
***AtGBSS***	−2.644	1.000	2.497	0.004	0.309	p > 0.10

*Z*-test of selection with null hypothesis (H_0_: dN = dS) was tested with two different alternative hypothesis such as positive selection (H_A_: dN > dS) and purifying selection (H_A_: dN < dS). *Z* statistics and Tajima’s *D* as well as significance values (p value) were calculated with coding sequences (CDS) of each starch synthase.

### Sequence comparison between *A*. *thaliana* and *A*. *lyrata*

*A*. *lyrata* is a close relative of *A*. *thaliana* and, therefore, permits the identification of putatively ancestral states of polymorphisms in *A*. *thaliana*. In Additional file 1: Figure S2, the nucleotide substitutions shared with *A*. *lyrata* are marked. The accession used as reference, Col-0, (*see above*) deviates frequently from *A*. *lyrata* but some *A*. *thaliana* accessions are more similar to *A*. *lyrata*. This is particularly evident in those genes forming two haplogroups (*AtSSI*, *AtSSIII*, *AtSSIV*, *AtGBSS*; cf. Figure 2, Additional file 1: Figure S2). In the most variable starch synthase, *AtSSIII*, more than half of the substitutions (25 out of 46) observed in C24, Can, Ct, and El also occur in *A*. *lyrata*. In addition, an indel consisting of seven amino acids in the D2 motif of the large N-terminal extension is present in C24, Can, Ct, El, and in *A*. *lyrata* as well but absent in Col-0 (Additional file 1: Figure S2D). In *AtSSIII*, there are particularly many sites where some *A*. *thaliana* accessions share a polymorphism with *A*. *lyrata*. However, the overall identity of their coding sequence is lower (0.953) as compared to Col-0 (0.976), because of fixed differences among the two *Arabidopsis* species. All starch synthase genes consistently show such a pattern of lower intra- (π) than interspecific variability (*K*) (Table 5).

**Table 5:** **Intra- and interspecific variation of starch synthases in *A. thaliana *and *A. lyrata***

				**Number of fixed differences**			**Number of polymorphic sites**		
	**π**	***K***	**π/*K *ratio**	**total**	**syn**	**nonsyn**	**total**	**syn**	**nonsyn**
***AtSSI***	0.0115	0.0152	0.7551	203	62	21	110	21	9
***AtSSII***	0.0014	0.0049	0.2895	162	52	38	30	6	13
***AtSSIII***	0.0056	0.0085	0.6580	175	53	31	106	30	34
***AtSSIV***	0.0028	0.0069	0.4128	274	87	43	68	14	17
***AtGBSS***	0.0045	0.0077	0.5822	134	37	14	49	16	12

Measures for complete gene sequences (exons and introns). π = nucleotide diversity within accessions of *A. thaliana*; *K =* nucleotide divergence between *A. thaliana* and *A. lyrata*; π/*K* = ratio of diversity and divergence. Number of fixed differences between *A. thaliana* and *A. lyrata*, as well as number of polymorphic sites among *A. thaliana* accessions, both subdivided into total, synonymous (syn), and nonsynonymous (nonsyn) substitutions.

### Analyses of promoter elements

In *A*. *thaliana*, expression of each starch synthase class has been reported to vary across plant organs and developmental stages [35-37]. *AtSSI* appears to be the major expressed isoform in roots, leaves, flowers, and immature fruits under long day conditions. By contrast, the expression of the other isoforms is lower according to the order *AtSSII* > *AtSSIV* > *AtSSIII*[35]. To determine polynucleotide substitutions within the promoter regions, we sequenced an approximately 1 kb large promoter region for each starch synthase. Subsequently, regulatory elements were identified by using the plant promoter database (PPDB; [38]).

For *AtSSI*, *AtSSIII*, and *AtSSIV*, there were no comparative data available for *cis*-regulatory elements in PPDB. In the promoter of *AtSSII*, a single area (-468 to -458; GTGGCCCAAAT) is described to contains four putative *cis*-regulatory elements (AtREG445: -468 to -461, AtREG420: -467 to -460, AtREG373: -466 to -459, AtREG421: -465 to -458). All elements are found in light-induced promoters (often designated as SORLIPs). In Ra, Rsch, Van, and Yo, this region (GTGGCCCAAAT) is affected by three substitutions and one deletion (leading to the sequence ----TCGATAT). In PPDB, two regions in *AtGBSS* (-234 to -247 and -446 to -453) are described containing conserved *cis*-regulatory elements. The former contains a bZIP-binding motif, while the function of the latter is unknown. Both regions are highly conserved. By using PPDB, we could identify a *cis*-regulatory element in *AtSSII* that appears to be involved in gene regulation. However, this assessment is preliminary, as PPDB does not provide full informations for the genes analyzed here.

### Expression analyses

Despite the fact we could not identify *cis*-regulatory elements in all starch synthase genes, we searched for differences in transcript levels among accessions and for correlations with haplotype clusters identified by respective gene trees. Based on the phylogenetic gene tree of the combined promoter and gene sequence, we selected six out of 30 accessions for each starch synthase. These accessions were selected such that they represent the different clusters identified for a particular gene (colored in Figure 2).

Transcript levels of starch synthases were tested for significant differences using one-way ANOVA. We found significant differences in expression levels across accessions for all starch synthases (*AtSSI*: 9.46∙10^-26^, *AtSSII*: 8.43∙10^-14^, *AtSSIII*: 2.58∙10^-4^, *AtSSIV*: 5.21∙10^-28^, *AtGBSS*: 4.88∙10^-23^). For the evaluation of pairwise differences in transcript levels, we used the post-hoc TukeyHSD test to estimate 95% confidence intervals. If these intervals do not include zero, transcript levels are significantly different in the respective pair of accessions (Figure 2).

Pairwise comparisons among 6 accessions revealed for *AtSSI* no differences in transcript levels between Gre, Sha, and Wil (Figure 2A), all of which assigned to cluster I in the gene tree. By contrast, accessions from cluster II showed significant differences in transcript levels in the order Edi < Bl < Can. In addition, their transcript levels also differed from cluster I accessions. For *AtSSII*, we identified two groups with similar transcript levels (group 1: Col, and Mt; group 2: Ct, Tsu, and Van; Figure 2B). Group 2 accessions had a significantly higher expression than group 1 accessions, while Can was intermediate. However, there was no correlation between gene tree and transcript levels. For *AtSSIII*, the transcript levels were similar among the accessions studied (Figure 2C), despite the occurrence of two distinct haplogroups (cluster I and II). The phylogenetic analysis of *AtSSIV* sequences revealed at least three haplogroups (Figure 2D). Accessions from cluster I (Er, Gre, and Ws) had similar transcript levels. The same holds true for accessions from cluster III (Can, and Cvi). Pairwise comparisons of either Can or Cvi (cluster III) and cluster I accessions revealed the most significant differences. The position of the analyzed accessions in the gene tree exactly correlates with the differences in the transcript levels among the analyzed accessions. In *AtGBSS*, transcript levels do not correlate with haplogroups (Figure 2E). Ct, Edi, Mt, and Rsch have similar transcript level, while Tsu and Van exhibit significantly higher expression levels.

## Discussion

Primary metabolism has been defined as ‘those essential reactions involving compounds that are formed as part of the normal anabolic and catabolic processes, which result in assimilation, respiration, transport, and differentiation processes that take place in most, if not all, cells of an organism’ [39]. It has been assumed that genes involved in primary metabolism are more conserved than secondary metabolism genes because of their essential function [40]. For this reason, gene-specific investigations of intraspecific variability among accessions of *A*. *thaliana* were largely performed with secondary metabolism genes [41-49]. Currently, numerous whole genomes become available as part of the 1,001 genomes project [50] become available. However, with these whole-genome approaches subtle differences within and across highly homologous gene loci can be overlooked and are easier detectable when targeting specific genes and their adjacent genomic regions [51]. Here, we demonstrate that genes encoding the starch synthases and exert essential functions in the plant primary metabolism possess high levels of nucleotide diversity as genes related to the secondary metabolism [41-49]. Furthermore, we show that transcript levels of starch synthases vary among accessions in all starch synthases, but both amount and pattern of variation differ between starch synthases. Variation was minor in *AtSSII*, *AtSSIII*, and *AtGBSS*, but higher in *AtSSI*, and *AtSSIV*. The differences in transcript levels clearly correlate with the gene tree in *AtSSIV*. We argue that such a strict correlation between combined promoter/gene sequences and transcript levels is indicative of *cis*-regulation. Such correlation was absence in *AtSSI*, *AtSSII*, *AtSSIII*, and *AtGBSS*, indicating *trans*-regulation is the major regulatory mechanism in these genes.

Obviously, functional analyses are expected to be more complete if the genetic variations were compared with the total starch synthase activity and/or the zymograms obtained for the various accessions. Unfortunately, this approach is not possible. Some starch synthases apparently contribute very little to the total enzyme activity measured in crude extracts. Furthermore, in zymograms performed with leaf extracts some AtSS isozymes are recovered as multiple bands but products of other AtSS genes are not detectable at all (although the respective recombinant proteins exhibit enzyme activity). Thus, zymograms do not reflect the genetic complexity of the starch synthases [52].

With regard to the specific pattern of variation, our study is able to address the following questions:

(i) Do genes of the five starch synthase classes exhibit a similar degree of both synonymous and nonsynonymous variation?

The five starch synthases exhibit different degrees of both synonymous and nonsynonymous variation. *AtSSI*, *AtSSIII*, and *AtGBSS* are the most variable genes exhibiting similar nucleotide diversities. *AtSSII* and *AtSSIV* possess the lowest nucleotide diversity. We confirm that *AtSSI* is highly diverse [29]. Presumably, nucleotide diversity correlates with the *in vivo* function of the protein. As outlined above, the five starch synthase classes are likely to exert non-identical *in vivo* functions. SSI to SSIII classes are involved in amylopectin biosynthesis, whereas GBSS is essential for the biosynthesis of amylose. AtSSIV appears to be essential in one route of the initiation of starch granule biosynthesis [30,31]. Mutants from *A*. *thaliana* lacking functional AtSSIV possess only a single, enlarged granule per plastid of the mesophyll cells [31]. Furthermore, overexpression of *AtSSIV* leads to increasing levels of both transitory and storage starch [32]. This specific function might be the reason for the lower nucleotide diversity in *AtSSIV*. Knockout mutants of *AtSSII* are deficient in intermediate α-glucan chain length with DP (degree of polymerization) of 12–25 [21]. This is compared to *AtSSI* (DP 8–12; [21]) and *AtSSIII* (DP 14–20; [53]) a broader spectrum. The lower level of genetic variation in *AtSSII* might be due to the partly overlapping function, by which AtSSII can substitute either AtSSI or AtSSIII. However, the reason for the relatively high level of nucleotide diversity in AtGBSS is not clear, as its ability to synthesize amylose is an important biological function [17].

(ii) Do specific gene trees of the five starch synthase classes show the same pattern of haplotype clustering across accessions?

One could assume that functionally (originated from an ancestral gene) related genes exhibit similar gene trees. In general, starch synthases show at least two haplogroups, except for *AtSSII*. The existence of diverse clusters of haplotypes has also been reported from Rubisco genes [51]. These divergent haplogroups are indicative of a relatively large long-term effective population size of the species and are likely to comprise ancient standing variation, rather than *in situ* divergence among accessions. As in Rubisco [51], the phylogenetic trees of the different starch synthase genes are neither congruent with one another nor do they reflect any geographical or ecological pattern. Because accessions are mainly homozygous inbred lines and, due to the local distance between naturally occurring populations, any exchange of gene variants between accessions is unlikely to occur. The incongruent phylogenetic pattern, however, could be indicative of relatively frequent recombination across accession (on an evolutionary timescale), by which gene tree and species/accession tree are disentangled. Only if many loci in combination or even whole genomes are phylogenetically analyzed, a reliable phylogeographic pattern can be detected among *A*. *thaliana* accessions [54,55].

(iii) Can selection be inferred to act on genes and/or single polymorphic sites?

The *Z*-tests revealed purifying selection is acting on *AtSSI*, *AtSSIII*, *AtSSIV*, and *AtGBSS*. Previous studies on *AtSSI* yielded significant positive values for Tajima’s *D*, interpreted as indication for balancing selection [29,56]. We also obtained positive (yet statistically not significant) *D* values for *AtSSI*. Because *A*. *thaliana* accessions comprise essentially homozygous inbred lines, we argue that such positive selection across accessions (as indicated by positive *D* values) should be interpreted as disruptive selection, i.e., divergent evolution among evolutionary lineages. As all the different accessions – because of their different geographic origin and a high level of selfing - very likely do not contribute to a single common gene pool, negative frequency-dependent (= balancing) selection across accessions (as postulated in [56]) appears less likely to us. Otherwise, a scenario of divergent evolution at *AtSSI* fits well the description of two haplotypes [56], a Col-0 type (designated as A type) and the Ler type (B type). According to our analysis, these groups are separated by 105 SNPs (thereof 21 in coding sequences) in *AtSSI*.

(iv) Do polymorphisms across accessions and starch synthase classes have functional implications?

Several nonsynonymous substitutions were found in each of the starch synthases, some of which located at positions that are highly conserved among plant species. For each starch synthase, several amino acids have been identified that are of particular functional importance [27,28,34,57]. Furthermore, we performed an interspecies comparison and searched for polymorphisms at sites that are involved in ADP-glucose binding as well as catalysis by generating an alignment including protein sequences of all available starch synthases from maize, rice, and *A*. *thaliana*. None of those highly conserved sites were substituted in any of the accessions analyzed in this study. During starch biosynthesis many enzyme activities closely cooperate and, therefore, any disturbance of this concerted action may result in complex alterations of the starch structure. Several starch-related enzymes are likely to undergo protein-protein interactions *in vivo* and, therefore, may exert their biochemical functions mainly (or exclusively) as constituent of a protein complex rather than as a single catalyst [58,59]. For heterotrophic tissues starch-related protein complexes have been described that consist of distinct starch synthases and branching enzyme (and the plastidial phosphorylase as well) and the formation of these high molecular weight complexes appears to be controlled by covalent protein modifications [58,59]. If a given enzyme is inefficient or even not functional due to amino acid substitutions, the resulting functional implications may reflect not only that of a single enzyme, but rather that of the respective protein complex. The lack of polymorphism at functionally crucial sites and the inferred pattern of purifying selection hence underline the functional importance and evolutionary conservation of these genes. It is highly unlikely that new mutation will be positively selected, although such mutations could – in theory - establish partly or completely novel complexes or functionalities.

The promoters of starch synthases exhibit numerous polymorphisms and indels, which potentially influence the transcript level. We found significant differences in transcript levels among accessions for each starch synthase. In *AtSSIV*, the only starch synthase gene inferred to be *cis*-regulated (see above), we found several positions in the promoter region which show a diversity pattern congruent with haplogroup assignment and transcript level. However, these motifs could not be assigned to any known regulatory element in the PPDB.

(v) Is there a relationship between genomic variation and transcript levels in starch synthases?

Analyses of differences in transcript levels between accessions and their position in the combined promoter and gene phylogenetic tree revealed different kinds of relationships. In *AtSSII*, *AtSSIII*, and *AtGBSS*, we were unable to detect a tight correlation between genetic variation and transcript levels. We propose that *trans*-regulation is the major actor in these genes, because the polymorphisms that are responsible for differences in gene expression are apparently far away from the gene [13,14]. By contrast, in *AtSSIV*, we found a clear correlation between genomic polymorphisms and transcript levels. For this reason, we propose *cis*-regulation as a major actor in *AtSSIV* expression [13,14]. *AtSSI* is somehow peculiar. We could not detect any differences in transcript levels among accessions representing haplotype cluster I. It seems that none of the polymorphisms found in the promoters of these accessions occurred in functionally relevant *cis*-elements. In contrast, in accessions of cluster II *cis*-regulatory elements might be affected by polymorphisms, because we could detect expression variation among them. We assume that both *cis*- as well as *trans*-regulation are active in the regulation of the expression of *AtSSI*.

AtSSI, AtSSII, and AtSSIII are important for building the amylopectin chains. They possess partly overlapping, but also individual properties in α-glucan chain elongation, while GBSS is mainly responsible for amylose synthesis [17,21,30,53,60].

## Conclusions

While screening 30 accessions of *A*. *thaliana* gene specifically, we detected several nonsynonymous substitutions in each of the five analyzed starch synthases (*AtSSI*, *AtSSII*, *AtSSIII*, *AtSSIV*, *AtGBSS*). Gene trees for single genes often revealed a clear-cut clustering of accessions, which is – however – not consistent across different starch synthase genes. Our results are compatible with previous findings that two haplogroups might adaptively (by positive selection) diverge in *AtSSI*. In general, we identified amino acid substitutions in the catalytic glucosyl transferase domains (GT5, GT1) in almost all enzymes. Some of these observed amino acid substitutions affect sites known to be highly conserved across different plant species. Transcript analyses revealed significant differences in all starch synthases, although the extent varies among them. Comparison of haplotype clustering and transcript levels of starch synthases is indicative of both *trans*- and *cis*-regulated genes. AtSSI, AtSSII, AtSSIII, and AtGBSS are important for chain elongation of amylopectin and amylose, respectively. The corresponding genes are mainly globally regulated by *trans*-regulation, whereby elongation of α-glucan chains might be coordinated in concert. AtSSIV has exclusive and far reaching function and a separate regulation by individual *cis*-regulatory transcription factors – as inferred by our analysis - appears reasonable.

## Methods

### Plant materials and cultivation

*Arabidopsis* accessions were kindly provided by Prof. Altmann (IPK Gatersleben, Germany). Prior to germination, seeds were kept for at least 2 days at 4°C. Seedlings and plants were grown in 1:1 (w/w) mixture of GS 90 soil and vermiculite.

For sequencing the plants were grown under controlled short day conditions (12 h light [120 μE m^-2^ s^-1^], 20°C; 12 h dark, 18°C). Leaves were harvested after four weeks. For Realtime experiments, plants were grown for four weeks under controlled long day conditions (16 h light [120 μE m^-2^ s^-1^], 21°C; 8 h dark, 18°C). Leaves at vegetative stage of the plants were harvested at middle of light period (after 8 h light).

### DNA isolation, PCR amplification and sequencing

Genomic DNA was extracted from a pool of leaves from three plants per accession using a modified CTAB procedure [61]. Primers for starch synthases *AtSSI* (*At5g24300*), *AtSSII* (*At3g01180*), *AtSSIII* (*At1g11720*), *AtSSIV* (*At4g18240*), and granule bound starch synthase *AtGBSS* (*At1g32900*) were designed based on the Col-0 sequence. For amplification and sequencing of the entire gene, primers were designed about 50 to 200 bp upstream and downstream the coding region. For analysis of the promoter region, primers were placed about 1.0 to 1.5 kb upstream the start codon. The fragments of 30 worldwide distributed accessions (An-2, Bl-1, Bsch-2, Bur-0, C24, Can-0, Cha-0, Col-0, Ct-1, Cvi-0, Edi-0, El-0, Er-0, Est-1, Gre-0, Ler-1, Mt-0, Nok-2, Oy-0, Ra-0, Rsch-0, Sap-0, Sha(kdara), Stw-0, Te-0, Tsu-1, Van-0, Wil, Ws-3, Yo-0) were amplified with the proof-reading polymerase Phusion (Finnzymes) and purified enzymatically by using Exonuclease I and Antarctic Phosphatase (New England Biolabs). The templates were directly used for sequencing on an ABI 3130xl automated sequencer (Applied Biosystems), using the BigDye® Terminator v3.1 Cycle Sequencing Kit (Applied Biosystems).

### RNA isolation, cDNA synthesis and realtime PCR

RNA was isolated with Invitrap® Spin Plant RNA Mini Kit (STRATEC Molecular) using the DCT lysis solution. For each accession, three independently isolated RNA preparations (three biological replicates) were performed and 2 ***μ***g were reversely transcribed using the RevertAid™ First Strand cDNA Syhthesis Kit (Fermentas). For each starch synthase, 6 out of 30 accessions were selected for Realtime experiments. Accessions were selected such that they represented all haplotype clusters identified in the maximum likelihood phylogenetic analysis with combined promoter and gene sequences. cDNA was used at 0.2 μl per Realtime-PCR run in a 10-μl reaction volume using SensiMix™ SYBR Low-ROX (Bioline) and a LightCycler® 480 (Roche). For each biological replicate, three technical replicates were performed. Expression was normalized to Ubiquitin (*UBC21*, *At5g25760*). Primer sequences were as follows: 5^′^-TTCACGTTACTTTGCCATGC-3^′^ and 5^′^-ACTTTGCGGCCAAAAGTATG-3^′^ for *AtSSI*, 5^′^-CCTGAATTTCGGCATCTGAG-3^′^ and 5^′^-AAGCCAAATTTCCATCACCA-3^′^ for *AtSSII*, 5^′^-CGGAATGGACAGGTTGTCTT-3^′^ and 5^′^-CCCCAGCATAAATCAAATGG-3^′^ for *AtSSIII*, 5^′^-CTGGCAAACAGCTTTTGTTG-3^′^ and 5^′^-TGATCCTGCATTCTGTCTGG-3^′^ for *AtSSIV*, 5^′^-CAAACGAGGAGTTGATCGTG-3^′^ and 5^′^-AACTGAACCGGAGTTGGTTG -3^′^ for *AtGBSS*, and 5^′^-CTGCGACTCAGGGAATCTTCTAA-3^′^ and 5^′^-TTGTGCCATTGAATTGAACCC-3′ for *UBC21*.

### Data analysis

#### Alignment

Sequences were assembled with BioEdit version 7.0.5 [62] and for each accession all variable sites were checked manually during the construction of a sequence contig. All sequences were manually aligned to the reference sequence of Col-0.

#### Estimation of nucleotide polymorphism

By using DnaSP version 5 [63], both intra- and interspecific analyses of nucleotide polymorphism were performed. For multidomain analyses we estimated the number of polymorphic sites (*S*), the total number of mutations (η), the number of insertions/deletions (indel), the number of haplotypes (*h*), haplotype diversity (*Hd*), nucleotide diversity (π), nucleotide divergence (*K*) between *A*. *thaliana* and *A*. *lyrata*, and the GC content, separately for promoters, exons, and introns (see [64] for diversity measures). *A*. *lyrata* sequences were obtained from the DOE Joint Genome Institute [65].

#### Evaluation of gene-wise selection

To test for natural selection, the frequencies of synonymous substitution per synonymous site (dS) were compared relative to those of nonsynonymous substitution per nonsynonymous site (dN), as implemented in MEGA version 4 [66]. The nonsynonymous to synonymous substitution rate ratio (ω) was calculated according to the modified model of Nei & Gojobori [67] with the correction of Jukes and Cantor [68] for saturation/multiple hits. With a *Z*-test, we assessed the likelihood of the null hypothesis of neutral evolution (H_0_: d_N_ = d_S_), relative to two alternatives, i.e., purifying selection (d_N_ < d_S_) and positive selection (d_N_ > d_S_). We also calculated Tajima’s *D*[69] which is an often used selection test based on the difference between two estimates of the amount of nucleotide variation. One estimate is obtained from the number of segregating sites [70] and the other is based on the average number of pairwise differences.

#### Selection at particular codons

Within a codon for a single amino acid, the ratio of nonsynonymous to synonymous substitution rate (ω) can be used for assessing selection, as values for ω <1, =1, and >1 are indicative of purifying selection, neutral evolution, and diversifying (= positive) selection, respectively. Positive selected sites (PSS), suggested by ω > 1, were searched for by using maximum-likelihood-based random-sites model analysis implemented in PAML 3.14 package [71,72]. For each starch synthase gene, analyses for each starch synthase gene were performed using run code “user tree” in codeml. The utilized maximum likelihood trees were constructed by RAxML 7.0.4 [73] under the GTR + G + I model with 1,000 bootstrap replicates. We performed one Likelihood Ratio Test (LRT) for positive selection (M7-8). M7 (beta) assumes a beta distribution of ω over sites, whereas model M8 (beta & ω) adds an additional site class (free ω ratio) which is estimated from the data set [72]. Occasions where the alternative model M8 is fitted better (p < 0.05) than the compared null model were considered as being positive selected.

#### Maximum likelihood gene tree

Using RAxML 7.0.4 [73] we constructed maximum likelihood gene trees for the combined promoter and gene data set of each starch synthase gene. The trees were generated under the GTR + G + I model of sequence evolution with 1,000 bootstrap replicates.

#### Promoter analyses

We sequenced the promoter region of about 1.0 kb for starch synthases to check if polymorphic sites affect ‘functionally important elements’ according to the plant promoter database PPDB [38] which we searched for regulatory elements and other important promoter regions, like TATA box.

#### Estimation of differences in expression levels among accessions

Expression levels were tested for significant differences in mean values among accessions using one-way ANOVA implemented in R [74]. In case of significant differences, the post-hoc pairwise comparison TukeyHSD test (implemented in R) was performed and confidence intervals of 95% were plotted.

## Competing interests

The authors declare that they have no competing interest.

## Authors’ contributions

SS performed the lab work as well as the bioinformatic analyses and drafted the manuscript. HB and MS participated in preparation of the manuscript and discussing the data from the starch metabolism point of view. RT supervised the study and participated in drafting the manuscript. All authors read and approved the final manuscript.

## Supplementary Material

Additional file 1: Figure S1 Position specific nucleotide diversity. Exons are marked grey; the regions containing exon sequences for the chloroplast transit peptied (cTP) as well as for the domains GT5 and GT1 are indicated. A: *AtSSI*; B: *AtSSII*; C: *AtSSIII*; D: *AtSSIV*; E: *AtGBSS*. **Figure S2.** Nucleotide polymorphisms in the coding sequence of starch synthases among *A*. *thaliana* accessions. Dots indicate identity to the reference Col-0. Nucleotide substitutions shared with *A*. *lyrata* are marked with asterisks above the position. Amino acid sustitutions are shown in the lower part of the column. The upper symbol indicates the amino acid in Col-0, while the lower is the substituted one. The GT5 and GT domains as well as the starch synthase III specific domains D1, D2, and D3 (see text) are highlighted in grey. A: *AtSSI*; B: *AtSSII*; C: *AtSSIII*; D: *AtSSIV*; E: *AtGBSS*. Click here for file
