# Supplementary material for: Intraspecific sequence variation and differential expression in starch synthase genes of Arabidopsis thaliana
Source: BMC Res Notes. 2013 Mar 6;6:84. doi: 10.1186/1756-0500-6-84 (PMC3608163; doi:10.1186/1756-0500-6-84)
Supplement: Additional file 1: Figure S1 — Position specific nucleotide diversity. Exons are marked grey; the regions containing exon sequences for the chloroplast transit peptied (cTP) as well as for the domains GT5 and GT1 are indicated. A: AtSSI; B: AtSSII; C: AtSSIII; D: AtSSIV; E: AtGBSS. Figure S2. Nucleotide polymorphisms in the coding sequence of starch synthases among A. thaliana accessions. Dots indicate identity to the reference Col-0. Nucleotide substitutions shared with A. lyrata are marked with asterisks above the position. Amino acid sustitutions are shown in the lower part of the column. The upper symbol indicates the amino acid in Col-0, while the lower is the substituted one. The GT5 and GT domains as well as the starch synthase III specific domains D1, D2, and D3 (see text) are highlighted in grey. A: AtSSI; B: AtSSII; C: AtSSIII; D: AtSSIV; E: AtGBSS. [file 1756-0500-6-84-S1.docx]

# Supplemental Figures

## Suppl. Figure 1 - Position specific nucleotide diversity.

## Exons are marked grey; the regions containing exon sequences for the chloroplast transit peptied (cTP) as well as for the domains GT5 and GT1 are indicated. A: *AtSSI*; B: *AtSSII*; C: *AtSSIII*; D: *AtSSIV*; E: *AtGBSS*.

## Suppl. Figure 2 - Nucleotide polymorphisms in the coding sequence of starch synthases among *A. thaliana* accessions.

Dots indicate identity to the reference Col‑0. Nucleotide substitutions shared with *A. lyrata* are marked with asterisks above the position. Amino acid sustitutions are shown in the lower part of the column. The upper symbol indicates the amino acid in Col-0, while the lower is the substituted one. The GT5 and GT domains as well as the starch synthase III specific domains D1, D2, and D3 (see text) are highlighted in grey. A: *AtSSI*; B: *AtSSII*; C: *AtSSIII*; D: *AtSSIV*; E: *AtGBSS*.

## Suppl. Figure 1 - Position specific nucleotide diversity.

**A *AtSSI***

##

**B *AtSSII***

**C *AtSSIII***

**D *AtSSIV***

**E *AtGBSS***

## Suppl. Figure 2 - Nucleotide polymorphisms in the coding sequence of starch synthases among *A. thaliana* accessions.

**A *AtSSI***

**B *AtSSII***

**C *AtSSIII***

**D *AtSSIV***

**E *AtGBSS***
